# Prognostic significance of controlling nutritional status in older adults with heart failure with preserved ejection fraction: a prospective comparative study with other objective nutritional indices

**DOI:** 10.1007/s40520-023-02395-x

**Published:** 2023-04-01

**Authors:** Ying Chen, Hui Zheng, Yu He

**Affiliations:** Department of Geriatrics, Beijing Tongren Hospital, Capital Medical University, Beijing, 100730 China

**Keywords:** Heart failure with preserved ejection fraction, Nutritional status control, Prognosis, Aged

## Abstract

**Objective:**

We explored the prognostic significance of controlling nutritional status (CONUT) score in older adults with heart failure with preserved ejection fraction (HFpEF) and compared CONUT with other objective nutritional indices.

**Methods:**

This is a single-center retrospective cohort study in older adult coronary artery disease patients undergoing HFpEF. Clinical data and laboratory results were collected before discharge. CONUT, geriatric nutritional risk index (GNRI), and prognostic nutritional index (PNI) were calculated according to the formula. The primary endpoint of this study was readmission due to heart failure and all-cause mortality in the first year after hospitalization.

**Results:**

A total of 371 older adults were enrolled. All patients were discharged and followed up for 1 year, and readmission for heart failure was 26% while all-cause mortality was 20%. Compared with the none and mild malnutrition risk group, the readmission rate for heart failure (HF) within 1 year (36% vs. 18%, 23%) and all-cause mortality rate in the moderate and severe malnutrition risk group (40% vs. 8%, 0%) were higher (*P* < 0.05). On multivariate logistic analysis, CONUT was not associated with readmission due to HF within 1 year. CONUT was significantly associated with all-cause mortality independently of GNRI or PNI, after adjustment for major confounders including age, bedridden; length of stay; history of chronic kidney disease; loop diuretics use; angiotensin-converting enzyme inhibitor/angiotensin receptor blocker and beta-adrenergic blocking agents use; New York Heart Association (NYHA) functional class; hemoglobin; potassium; Creatinine; triglycerides; glycosylated hemoglobin; brain natriuretic peptide; left ventricular ejection fraction; GNRI and PNI via multivariable Cox analysis (HR (95% CI) 1.764 (1.503, 2.071); 1.646 (1.359, 1.992); 1.764 (1.503, 2.071), respectively). Kaplan–Meier analysis revealed that the risk of all-cause mortality significantly increased in accordance with a higher CONUT (CONUT 5–12 compare to 0–1:HR (95% CI) 6.16 (3.78, 10.06); CONUT 2–4 compare to 0–1:HR (95% CI) 0.16 (0.10, 0.26)). CONUT showed the best area under the curve value (0.789) for the prediction of all-cause mortality compared with the other objective nutritional indices.

**Conclusion:**

CONUT is a simple and strong prognostic indicator for the prediction of all-cause mortality in older adults with HFpEF.

**Clinical Trials.gov Identifier:**

NCT05586828.

## Introduction

Heart failure (HF) is an advanced terminal stage of various heart diseases, with high rehospitalization and mortality rates [1, 2]. With the aging of population and the improvement of medical care, the prevalence of HF has increased rapidly, which contributed to a growing health burden worldwide. The proportion of older adults with HF will further increase [3]. In HF patients, malnutrition is not uncommon and represents one of the most significant determinants of poor clinical outcomes. Cardiac cachexia, an involuntary non-edematous weight loss within 6–12 months, is observed in 5–20% of patients with chronic HF and is associated with poor prognosis [4–7]. Therefore, nutritional management is of paramount importance for patients with HF.

The basic method of nutritional management is to effectively identify the nutritional status of patients with heart failure, especially in older adults with heart failure who are not easy to be found in the early stage. CONUT has been shown to be associated with poor prognosis in various cardiovascular areas, especially in patients with acute decompensated heart failure [8, 9]. However, limited data are available regarding the association between CONUT and prognosis in elder patients with HFpEF, despite its easy availability in routine blood chemistry. On the other hand, the prognostic value of various objective nutritional indices, such as GNRI and PNI has been reported recently in HF patients. There is few information available comparing the prognostic utility of CONUT with that of the other established nutritional indices in elder patients with HFpEF.

Therefore, we aimed to evaluate the prognostic significance of CONUT and to compare it with other well-established nutritional indices in HFpEF, a common HF phenotype in older adults with coronary artery disease.

## Materials and methods

### Study population

**Fig. 1:**
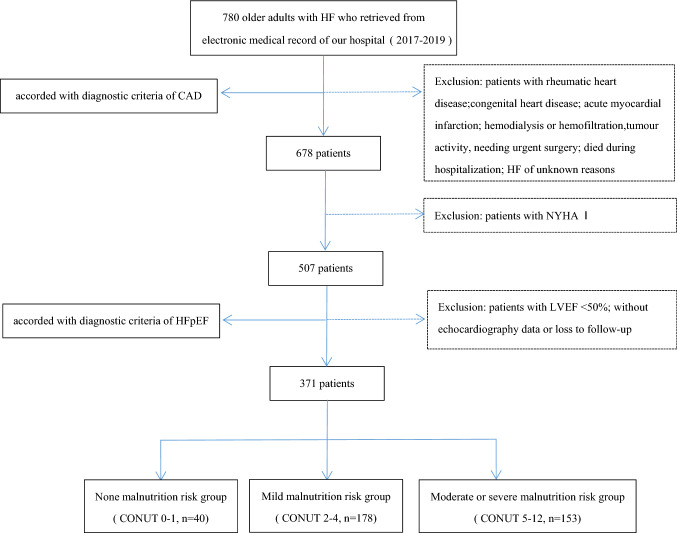
Flow of patients through the study. *CAD* coronary artery disease, *HF* heart failure, *NYHA* New York Heart Association, *LVEF* left ventricular ejection fraction, *CONUT* controlling nutritional status

This was a single-center retrospective cohort study. The reason for the admission of enrolled patients was an aggravation of cardiac insufficiency. The flow of patients through the study is shown in Fig. 1. Clinical Trials.gov Identifier: NCT05586828. This study was approved by the Clinical Research Ethics Committee of Beijing Tongren Hospital, Capital Medical University (TRECKY2021-185). Written informed consent was obtained from all patients.

Coronary artery disease was defined as at least 1 of the following: (1) definite diagnosis by coronary angiography or coronary computed tomography (≥ 50% stenosis of the internal diameter of at least 1 or more major coronary arteries or branches); (2) previous history of old myocardial infarction, abnormal Q waves in 2 consecutive leads of electrocardiogram, and or previous biochemical markers of myocardial necrosis elevated, and or previous interventional therapy or surgical coronary revascularization. The diagnosis of HF was made according to the recommendations of the European Society of Cardiology [10]. HFpEF was defined as follows [11]: (1) dyspnea, fatigue, or decreased activity tolerance; (2) signs of fluid retention (pulmonary stasis and peripheral edema); (3) echocardiography showing left ventricular ejection fraction (LVEF) ≥ 50% on echocardiography and at least 1 of the following: (1) left ventricular hypertrophy and/or left atrial enlargement; (2) abnormal diastolic function; (4) B-type brain natriuretic peptide (BNP) elevated. The severity of functional class was based on NYHA scale.

### Data collection

Demographic characteristics, medical parameters, such as age, gender, body mass index (BMI), length of stay, bedridden, NYHA class, and comorbidities, were collected from the electronic medical record system. Laboratory data were obtained from the clinical chemistry department within 1 week before discharge including hemoglobin, lymphocytes, potassium, sodium, fasting blood glucose, creatinine, total protein, albumin, triglycerides, total cholesterol, low-density lipoprotein cholesterol, high-density lipoprotein cholesterol, glycated hemoglobin, brain natriuretic peptide, systolic blood pressure, heart rate, left ventricular ejection fraction. Discharge medication usage including angiotensin-converting enzyme inhibitor (ACEI), angiotensin receptor blocker (ARB), beta-adrenergic blocking agents (β-blocker), mineralocorticoid-receptor antagonists (MRA), loop diuretics and statin were also recorded. The CONUT was calculated from 3 variables: serum albumin concentration, total cholesterol concentration, and lymphocyte count, as previously reported [12, 13]. Total scores of 0–1 are health, and scores of 2–4, 5–8, and 9–12, respectively, indicate mild, moderate, and severe malnutrition risk. We also assessed the patient nutritional status by GNRI [14, 15] and PNI [16, 17]. GNRI = 1.489 × serum albumin (g/L) + 41.7 × BMI/22. GNRI > 98 is health, 92 ≤ GNRI ≤ 98, 82 ≤ GNRI < 92, and GNRI < 82, respectively, indicate mild, moderate, and severe malnutrition risk. PNI = serum albumin (g/L) + 5 × total lymphocyte count (10^9^/L). PNI ≥ 50 is health, 45 ≤ PNI < 50, 40 ≤ PNI < 45, PNI < 40, respectively, indicate mild, moderate, and severe malnutrition risk. BMI is defined as body weight divided by the square of height.

### Clinical outcomes and follow up

After discharge, all enrolled patients were followed-up in an outpatient setting. Survival data were obtained via direct contact with patients or patients’ caregiver by their physicians at the hospital, or via telephone interviews of their families by dedicated coordinators and investigators. The follow-up lasted for one year and the last follow-up ended on December 31, 2020. The primary endpoint was readmission due to heart failure and all-cause mortality in the first year after hospitalization.

### Statistical analysis

Continuous measurement data with or without a normal distribution were expressed as the means ± SDs or medians with interquartile range (IQR). Normal distributional data were compared between three groups using analysis of variance. The rank sum test was used to compare differences in non-normal distribution data. Categorical data were expressed as frequencies and percentages and compared by the Chi-square. Multivariate logistic analysis was performed to identify the indicators for readmission in one year after discharge. Cox proportional hazards regression models were used to identify patients at risk of all-cause mortality to calculate the multivariable-adjusted HRs and 95% CIs. Three models were constructed: model 1, fully adjusted for major potential confounders; model 2, adjusted for major potential confounders plus GNRI; model 3, adjusted for major potential confounders plus PNI. Kaplan–Meier survival curves and a log-rank test were used to compare mortality among three risk-stratified groups. A receiver operating characteristic (ROC) curve was constructed using CONUT, GNRI, and PNI as the test variables and readmission and all-cause death events as the state variables. The area under the ROC curves (AUCs) was used to compare the predictive value of CONUT, GNRI, and PNI. All analyses were performed using SPSS version 19.0 (SPSS, Chicago, IL, USA). A *p* value of < 0.05 was considered to be statistically significant.

## Results

### Baseline patient characteristics

The median value of CONUT was 4. The study population was categorized by CONUT as follows: CONUT 0–1, none malnutrition risk group (*n* = 40), CONUT 2–4, low malnutrition risk group (*n* = 178), CONUT 5–12, mild and high malnutrition risk group (*n* = 153). Baseline characteristics of 371 older adult coronary artery disease patients with HFpEF were stratified by CONUT and are shown in Table 1. The mean age was 88 years and 70% were male, and 81% were over 80 years old. Since 19% of the patients were bedridden, BMI and GNRI could only be evaluated in 302 patients. The average length of stay was 12 days. NYHA class on discharge was II in 52% of patients, III in 38% of patients, and IV in 10% of patients. All patients were discharged and followed up for 1 year, readmission for heart failure was 26% while all-cause mortality was 20%.

**Table 1:** Baseline characteristics in older adults with coronary artery disease heart failure preserved ejection fraction grouped by CONUT^1^

	ALL	0 ≤ CONUT ≤ 1	2 ≤ CONUT ≤ 4	CONUT ≥ 5	*p* value
	*n* = 371	*n* = 40	*n* = 178	*n* = 153	
*Clinical data*					
Age, y	88 (83–91)	84 (78–90)	86 (81–90)	89 (85–92)	< 0.001^b^
> 80,%	81	63	77	90	< 0.001^ab^
Gender (male), %	70	45	68	79	< 0.001^ab^
BMI^2^, kg/m^2^	24.0 ± 3.9	25.7 ± 3.0	24.3 ± 3.8	23.0 ± 4.0	< 0.001^ab^
Bedridden,%	19	10	9	32	< 0.001^b^
Length of stay, d	12 (9–15)	10 (7–12)	11 (8–14)	14 (11–19)	< 0.001^b^
NYHA class II /III/IV, %	52/38/10	75/23/3	62/30/8	35/51/14	< 0.001^b^
SBP, mmHg	130 (120–137)	130 (120–140)	130 (120–137)	128 (120–137)	0.532
HR, bpm	70 (64–74)	68 (63–72)	69 (64–74)	72 (66–77)	0.001^b^
*Comorbidities*,%					
OMI	26	20	21	32	0.059^b^
PCI	18	25	17	18	0.485
CABG	9	5	8	11	0.385
AF	37	30	34	42	0.230
HT	85	88	85	86	0.908
DM	62	58	63	61	0.812
COPD	25	23	22	28	0.406
CKD	36	10	35	43	< 0.001^a^
Tumor	16	3	16	19	0.039^a^
Dementia	16	8	15	20	0.109
*Medications*,%					
ACEI/ARB	42	53	49	30	0.001^b^
β-blocker	60	55	61	61	0.755
MAR	32	20	28	41	0.011^b^
Loop diuretics	35	20	26	48	< 0.001^b^
Statin	78	88	81	73	0.059^b^
ACEI/ARB + β-blocker	27	30	34	18	0.006^b^
*Laboratory data*					
Hb, g/L	122 (105–132)	133 (123–141)	124 (111–133)	110 (97–127)	< 0.001^ab^
Lyc, *10^9^/L	1.4 (1.1–1.8)	2.0 (1.6–2.4)	1.4 (1.1–1.8)	1.2 (0.9–1.5)	< 0.001^ab^
K, mmol/L	4.1 ± 0.4	4.0 ± 0.3	4.1 ± 0.4	4.1 ± 0.4	0.582
Na, mmol/L	139.2 (137.0–141.1)	140.0 (138.0–141.0)	140.0 (137.9–142.0)	139.0 (136.1–141.0)	0.060^b^
Glu, mmol/L	5.6 (4.8–6.8)	5.6 (4.9–7.1)	5.7 (4.9–6.8)	5.4 (4.7–6.7)	0.127
Cr, µmol/L	87.6 (70.9–111.0)	76.9 (66.1–91.4)	86.0 (71.0–107.7)	93.8 (72.8–127.1)	0.003^a^
TP, g/L	62.6 ± 6.5	67.7 ± 4.9	63.6 ± 5.1	60.1 ± 7.2	< 0.001^ab^
Alb, g/L	34.7 ± 4.3	38.6 ± 2.7	36.7 ± 3.2	31.4 ± 3.3	< 0.001^ab^
TG, mmol/L	1.0 (0.7–1.4)	1.5 (1.0–2.0)	1.1 (0.8–1.4)	0.7 (0.6–1.0)	< 0.001^ab^
TC, mmol/L	3.5 (3.0–4.2)	4.8 (4.0–5.5)	3.6 (3.1–4.4)	3.2 (2.7–3.7)	< 0.001^ab^
LDL-C, mmol/L	1.9 (1.5–2.4)	3.0 (2.3–3.5)	1.94 (1.5–2.5)	1.7 (1.3–2.0)	< 0.001^ab^
HDL-C, mmol/L	1.1 (0.8–1.3)	1.1 (0.9–1.5)	1.1 (0.9–1.3)	1.0 (0.8,1.2)	0.082
HbA1c, %	6.3 (5.8–7.2)	6.5 (6.0–7.9)	6.4 (5.9–7.3)	6.1 (5.7–7.0)	0.079
BNP, mmol/L	188 (94–387)	136 (68–235)	158 (78–296)	310 (131–577)	< 0.001^b^
*Echocardiography*					
LVEF, %	61 (57–65)	61 (58–64)	62 (57–64)	60 (55–65)	0.154
*Other nutritional indices*					
GNRI^2^	97 ± 11	106 ± 7	101 ± 9	89 ± 11	< 0.001^ab^
PNI	42 ± 6	49 ± 4	44 ± 4	38 ± 4	< 0.001^ab^
*Outcome*					
Readmission,%	26	23	18	36	0.001^b^
All-cause death,%	20	0	8	40	< 0.001^b^

^1^Differences between CONUT groups were tested by 1-factor ANOVA for normally distributed continuous variables. The Kruskal–Wallis rank sum test was used to compare differences in non-normally distributed data. Fisher’s exact test and the chi-square test were used to compare differences in categorical variables

*CONUT* controlling nutritional status, *BMI *body mass index, *NYHA* New York Heart Association, *SBP* systolic blood pressure, *HR *heart rate, *bpm* beats per minute, *OMI* old myocardial infarction, *PCI* percutaneous coronary intervention, *CABG* coronary artery bypass grafting, *AF* atrial fibrillation, *HT* hypertension, *DM* diabetes mellitus, *COPD* chronic obstructive pulmonary disease, *CKD* chronic kidney disease, *ACEI* angiotensin-converting enzyme inhibitor, *ARB* angiotensin receptor blocker, *β-blocker *beta-adrenergic blocking agents, *MRA* mineralocorticoid-receptor antagonists, *Hb* hemoglobin, *Lyc* lymphocytes, *K* potassium, *Na* sodium, *Glu* Fasting blood glucose, *Cr* Creatinine, *TP* total protein, *Alb* albumin, *TG* triglycerides, *TC *total cholesterol, *LDL-C* Low-density lipoprotein cholesterol, *HDL-C* high-density lipoprotein cholesterol, *HbA1c* glycosylated hemoglobin, *BNP* brain natriuretic peptide, *LVEF* left ventricular ejection fraction, *GNRI* geriatric nutritional risk index, *PNI* prognostic nutritional index

^2^ BMI and GNRI were evaluated in 302 patients due to bedridden

*p* value for differences among three groups by CONUT. ^a^2 ≤ CONUT ≤ 4 VS. 0 ≤ CONUT ≤ 1, *p* < 0.05; ^b^CONUT ≥ 5 VS. 2 ≤ CONUT ≤ 4, *p* < 0.05

### The comparison of baseline parameters by CONUT

Patients in the mild- and high-risk stratification had a higher age and bedridden, lower BMI, a longer length of stay, higher NYHA class and heart rate, higher patients with chronic kidney disease, lower use rate of ACEI/ARB and β-blocker, lower hemoglobin, lymphocyte, total protein, albumin, triglyceride, total cholesterol, and low-density lipoprotein concentrations, a higher creatinine and BNP concentration, higher incidence of readmission for heart failure and all-cause death in one year on discharge (all *P* < 0.05).

### Clinical outcomes and prognostic analysis

All patients were discharged and followed up for 1 year, readmission for heart failure was 26% while all-cause mortality was 20%. Univariable logistic analysis revealed that bedridden, NYHA III and IV, ACEI/ARB + β-blocker, length of stay, hemoglobin, creatinine, triglycerides, BNP, LVEF, CONUT, GNRI, and PNI were significantly associated with readmission for heart failure. Multivariable logistic analysis revealed that CONUT was not associated with readmission for heart failure after adjustment for major confounders (Table 2).

**Table 2:** Multivariable logistic analysis for the prediction of readmission due to heart failure within 1 year ^1^

Characteristic	Univariate		Multivariate	
	HR (95%CI)	*p* value	HR (95%CI)	*p* value
Age	1.034(0.998–1.071)	0.066		
Bedridden (ref = no)				
Yes	1.849 (1.058–3.231)	0.031		
NYHA (Ref = II)				
III	1.914 (1.150–3.185)	0.013		
IV	3.707 (1.767–7.778)	0.001		
CKD (Ref = no)				
Yes	1.599 (0.994–2.572)	0.053		
ACEI/ARB + β-blocker (Ref = no)				
Yes	2.416 (1.316–4.435)	0.004		
Length of stay	1.028 (1.001–1.056)	0.044		
Hb	0.979 (0.967–0.992)	0.002		
Cr	1.008 (1.004–1.012)	< 0.001	1.008 (1.003–1.014)	0.005
TG	0.630 (0.411–0.967)	0.035		
BNP	1.001 (1.000–1.001)	0.006	1.001 (1.000–1.002)	0.015
LVEF	0.937 (0.891–0.985)	0.010		
CONUTscore	1.230 (1.115–1.358)	< 0.001		
GNRI^2^	0.973 (0.950–0.996)	0.023		
PNI	0.930 (0.890–0.972)	0.001		

^1^Univariate and multivariate analyses were performed using logistic analysis to identify meaningful prognostic factors

*NYHA* New York Heart Association, *HR* heart rate, *CKD *Chronic kidney disease, *ACEI* angiotensin-converting enzyme inhibitor, *ARB* angiotensin receptor blocker, *β-blocker* beta-adrenergic blocking agents, *Hb* hemoglobin, *Glu* Fasting blood glucose, *Cr* Creatinine, *TG* triglyceride, *BNP* brain natriuretic peptide, *LVEF* left ventricular ejection fraction, *CONUT* controlling nutritional status, *GNRI* geriatric nutritional risk index, *PNI* prognostic nutritional index

^2^GNRI was evaluated in 302 patients due to bedridden

Univariable Cox proportional hazards analysis revealed that age, bedridden, NYHA III and IV, ACEI/ARB + β-blocker, loop diuretics, length of stay, hemoglobin, BNP, LVEF, CONUT, GNRI, and PNI were significantly associated with all-cause mortality (Table 3). Multivariable Cox proportional hazards analysis revealed that CONUT (HR 1.764, 95%CI 1.503–2.071, *P* < 0.001) was significantly independently associated with all-cause mortality after adjustment for major confounders (Table 4, model 1). Even after adjustment for major confounders in models including other objective nutritional indices, CONUT (HR 1.646, 95%CI 1.359–1.992, *P* < 0.001 and HR 1.764, 95%CI 1.503–2.071, *P* < 0.001 respectively) was still significantly independently associated with all-cause mortality (Table 4, models 2, 3).

**Table 3:** Univariable Cox analysis for the prediction of all-cause mortality^1^

Variables	HR (95% CI)	*p* value
Age	1.061 (1.022–1.102)	0.002
Bedridden (Ref = no)		
Yes	0.294 (0.185–0.468)	< 0.001
NYHA (Ref = II)		
III	0.219 (0.114–0.420)	< 0.001
IV	0.506 (0.277–0.922)	0.026
ACEI/ARB + β-blocker (Ref = no)		
Yes	1.861 (1.024–3.384)	0.042
Loop diuretics (Ref = no)	0.619 (0.392–0.977)	0.039
Length of stay	1.046 (1.032–1.059)	< 0.001
Hb	0.970 (0.958–0.982)	< 0.001
Cr	1.002 (1.000–1.005)	0.053
TG	0.674 (0.435–1.043)	0.077
BNP	1.001 (1.000–1.001)	< 0.001
LVEF	0.934 (0.889–0.982)	0.007
CONUTscore	1.555 (1.419–1.705)	< 0.001
GNRI	0.949 (0.927–0.971)	< 0.001
PNI	0.862 (0.824–0.902)	< 0.001

^1^Univariate analysis was performed using Cox’s regression model to identify meaningful prognostic factors

*NYHA* New York Heart Association, *ACEI* angiotensin-converting enzyme inhibitor, *ARB* angiotensin receptor blocker, *β-blocker* beta-adrenergic blocking agents, *Hb* hemoglobin, *Cr* Creatinine, *TG* triglyceride, *BNP* brain natriuretic peptide, *LVEF* left ventricular ejection fraction, *CONUT* controlling nutritional status, *GNRI* geriatric nutritional risk index, *PNI* prognostic nutritional index

^2^GNRI was evaluated in 302 patients due to BMI

**Table 4:** Cox multivariable proportional hazard models for the prediction of all-cause mortality^1^

	Model 1^2^-fully adjusted		Model 2^3^-fully adjusted + GNRI		Model 3^3^-fully adjusted + PNI	
	HR (95% CI)	*P* value	HR (95% CI)	*P* value	HR (95% CI)	*P* value
CONUTscore	1.764 (1.503, 2.071)	< 0.001	1.646 (1.359, 1.992)	< 0.001	1.764 (1.503, 2.071)	< 0.001

^1^Multivariate analysis was performed using Cox’s regression model to identify meaningful prognostic factors

*CONUT* controlling nutritional status, *GNRI* geriatric nutritional risk index, *PNI* prognostic nutritional index

^2^Cox multivariable proportional hazards regression analysis was used in model 1 to adjust for age, bedridden; length of stay; history of chronic kidney disease; loop diuretics use; angiotensin-converting enzyme inhibitor/angiotensin receptor blocker + beta-adrenergic blocking agents use; NYHA; hemoglobin; potassium; Creatinine; triglycerides; glycosylated hemoglobin; brain natriuretic peptide; left ventricular ejection fraction

^3^Cox multivariable proportional hazards regression analysis was used in models 2 and 3. Models 2 and 3 included the model 1 confounders plus GNRI and PNI, respectively

**Fig. 2:**
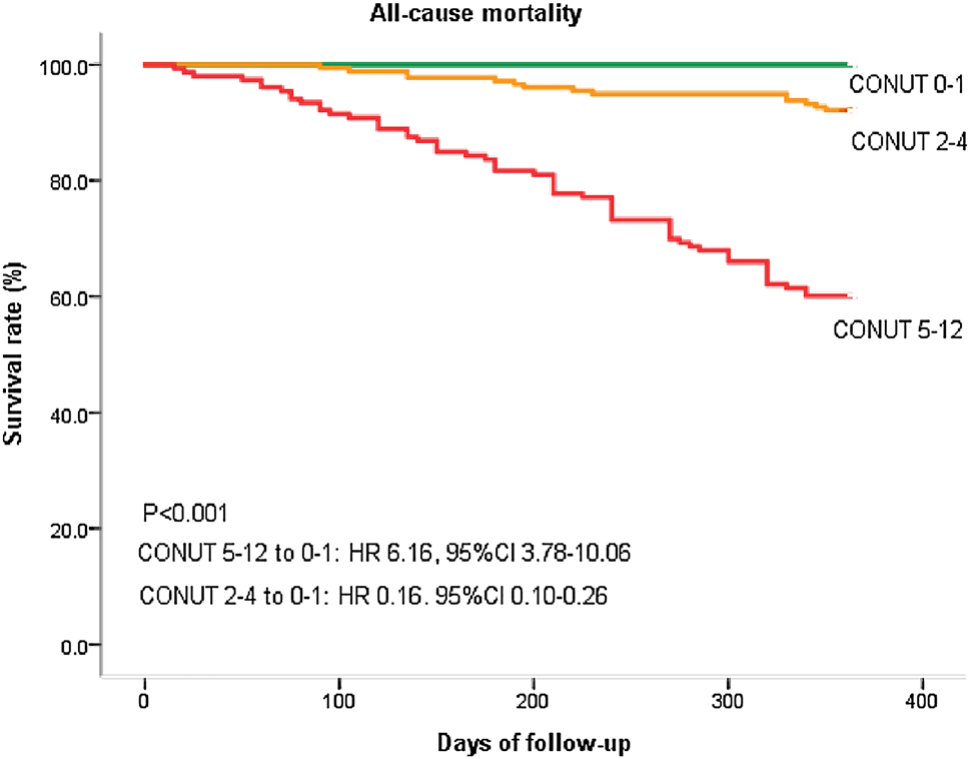
Kaplan–Meier estimates of all-cause mortality with patients stratified by different CONUT groups

Kaplan–Meier analysis revealed that the risk of all-cause mortality significantly increased in accordance with CONUT. Patients in the CONUT 5–12 group (HR 6.16, 95% CI 3.78–10.06, *P* < 0.001) had significantly greater risks of all-cause mortality compared with the CONUT 0–1 group (Fig. 2).

### Comparison with other nutritional indices

**Fig. 3:**
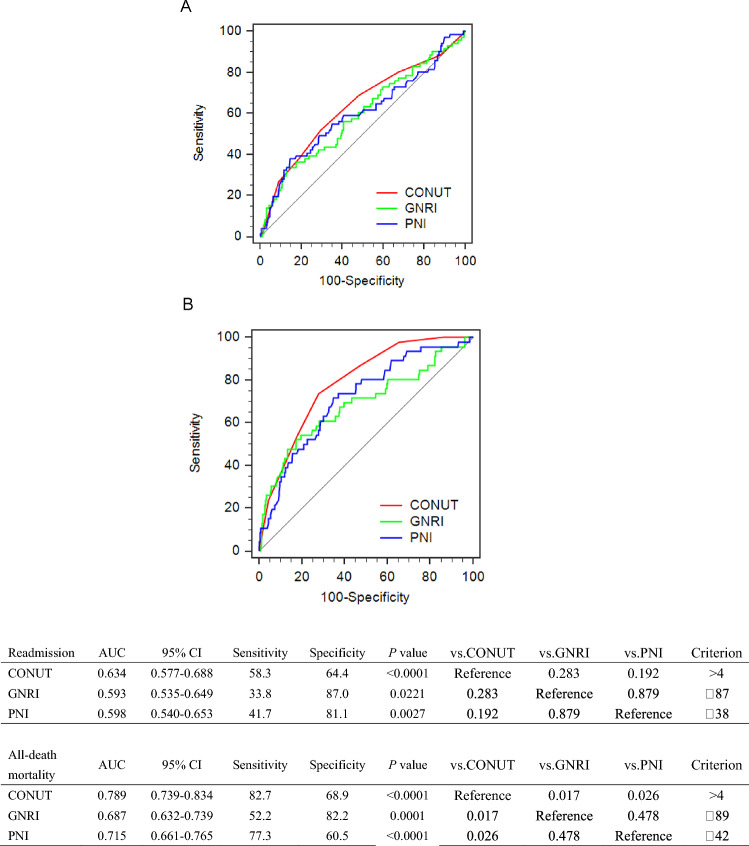
Receiver operating characteristic curves and comparison of predictive values for incidence of readmission for heart failure (**A**) and all-cause death (**B**) in one year on discharge. *CONUT* controlling nutritional status, *GNRI* geriatric nutritional risk index, *PNI* prognostic nutritional index

With the incidence of readmission for heart failure and all-cause death in one year on discharge as an endpoint, AUC was performed to compare the predictive ability of CONUT with that of other objective nutritional indices (Figs. 2, 3). CONUT exhibited AUC for incidence of readmission for heart failure in one year on discharge with no significant difference when compared with GNRI and PNI (*p* > 0.05).

CONUT exhibited a greater AUC for all-cause death in one year on discharge (AUC 0.826) than GNRI (AUC 0.687) and PNI (AUC 0.729) with a significant difference when compared with GNRI (*p* = 0.030) and with PNI (*p* = 0.0006) respectively (Fig. 3).

## Discussion

In this single-center retrospective cohort study among older adults undergoing HFpEF, we found that CONUT was a simple, strong prognostic marker for the prediction of all-cause mortality, even when compared with other objective nutritional indices.

The CONUT is calculated based on serum albumin, total cholesterol, and peripheral blood lymphocyte count. The content of serum albumin and cholesterol is the main index to measure patients' malnutrition, and the lymphocyte level reflects the body's immune function to a certain extent [8, 18, 19]. The pathophysiology of malnutrition in patients with heart failure is not clear. The underlying pathophysiological mechanism between malnutrition and heart failure can be explained in two ways [20, 21]. One is that fluid retention leads to intestinal edema, nausea, anorexia, and other symptoms, affecting the intake and absorption of nutrients. The second possibility is that the changes in intestinal morphology and function destroy the immune barrier of the intestinal wall and trigger the release of pro-inflammatory cytokines. Chronic inflammation and neurohormone activation also promote catabolism, leading to protein and adipose tissue degradation, leading to weight loss and cachexia. In recent years, among patients with heart failure, the predictive effect of CONUT on malnutrition has received extensive attention [9, 22, 23]. Each item of CONUT indicates the status of protein reserve consumption, calorie consumption, and immune defense damage, respectively. The combination of inflammation and nutritional status can improve the predictive significance, which may be the reason why the predictive value of CONUT is better than other malnutrition screening indicators.

With the aggravation of China's aging population, older adults with heart failure will further increase. At present, China's largest heart failure registration research "China heart failure center registration research" showed that older adults with heart failure ≥ 80 years old accounted for 21.8% [24, 25]. HFpEF is more common in older adults. However, due to the particularity of the diagnosis, evaluation, and treatment of older adults with heart failure, many clinical studies exclude older adults from the trial population, so it is necessary to further study [26, 27].

In this study, 81% of selected participants were over 80 years old, and we further compared the prognostic utility between CONUT and two other nutritional evaluation indexes in this old HFpEF cohort. GNRI is a nutritional evaluation index based on serum albumin and BMI. Bouillanne et al. [14] first proposed GNRI in 2005 to predict malnutrition-related complications (bedsore and infection) and mortality in hospitalized older adults. Minamisawa, et al. selected HFpEF patients from TOPCAT (aldosterone antagonist therapy for adults with heart failure and preserved systolic function) study and grouped them according to GNRI. About one-third of the patients took the risk of malnutrition (11% for moderate to severe nutrition, 25% for low nutrition, and 64% for no nutrition). During the 2.9-year follow-up, the risk of moderate to severe malnutrition was significantly associated with an increased risk of cardiovascular death, hospitalization for heart failure, and all-cause death compared with patients without malnutrition risk (HR 1.34, 95% CI 1.02–1.76; HR 2.06, 95% CI 1.79; 1.40–3.03; HR 1.79, 95% CI 1.33–2.42, respectively) [28]. Different from the above results, this study did not show the prediction of GNRI on the risk of readmission or all-cause death in one-year follow-up. The prediction ability of CONUT was stronger than that of GNRI in all-cause death. The main reasons for this were as follows: The average age of this study and the proportion of old-old patients were considerably high. 69 older adults of all participants accounting for 18.6% were bedridden. Due to the inability to obtain accurate height and weight, their BMI was missing. Even among the old adults who were not bedridden, some had difficulty in accurately measuring their height and dry weight due to kyphosis or more fluid load in their bodies; some had both malnutrition and obesity [29]. As a result, the prediction ability of CONUT was better than that of GNRI calculated based on BMI in older adults with HFpEF.

PNI is a composite index that can reflect the nutritional status and immune status at the same time based on serum albumin level and peripheral blood lymphocyte count, which is mostly used for the prognosis of postoperative surgery or tumor patients. It does not involve anthropometric parameters, so it is also included in this study, but PNI fails to enter the equation after multifactor analysis. Compared with PNI, in addition to the common parameters of albumin and lymphocytes, CONUT also includes total cholesterol. Hypercholesterolemia is a well-recognized risk factor for cardiovascular morbidity and mortality in the general population, but this association seems to reverse with aging. Curciof, et al. [30] reviewed a lot of research and proposed that low BMI, blood pressure, and cholesterol values were associated with a worse prognosis in the elderly patient. He postulated a new phenotype identified as “catabolic syndrome” and discussed their possible mechanisms. Older patients with chronic disease states may not survive enough to die from long-term over-nutrition since they are more likely to die earlier from under-nutrition state and do not live long enough to have the negative long-term consequences of cardiovascular disease risk factor, which is the differences in nutritional risk factors of the older population compared to the younger one. It may also be the reason why CONUT is superior to PNI as a nutritional status assessment tool in older adults with HFpEF.

In fact, many nutrition screening tools have been developed in recent years, such as nutrition risk screening 2002, mini nutrition assessment short form, subjective global assessment, etc., which are characterized by simplicity and frequently used in the comprehensive assessment of older adults. However, there are many items of screening tools collected in the form of scales, which are greatly affected by patients' subjective factors and cognitive disorders, so they are not suitable for older adults, especially older patients with heart failure.

Chronic heart failure is a common condition among elderly persons. The prevalence of heart failure increases sharply with age. This increase is mainly due to the implementation of more effective treatment methods, which have improved the life expectancy of patients with heart failure. Therefore, more patients older than 65 experience clinical problems, including frailty and sarcopenia which were never traditionally considered as a relevant issue but have become a major clinical challenge for physicians nowadays.

The relationship between frailty, sarcopenia, and chronic heart failure is very complex as chronic heart failure may lead to frailty and frail patients may be at greater risk of developing chronic heart failure [31, 32], heart failure may induce sarcopenia, and sarcopenia may favor heart failure development by different mechanisms [33]. As frailty, sarcopenia and heart failure appear to share several pathogenetic pathways, the assessment of early nutritional status seems particularly important.

CONUT is an objective and easily available biomarker. Compared with other nutritional indicators, CONUT can provide more powerful prognostic information for older HFpEF patients. This indicator may be used as a more accurate nutritional assessment for older patients with heart failure, so that we can implement an early and appropriate nutritional intervention in daily practice, and bring better prognosis for older patients with heart failure.

Despite these strengths, several study limitations are worth noting. First, this was a single-center, retrospective and observational study, which was subject to the clinical data of patients to a certain extent. The sample size of this study was limited, and the follow-up time was relatively short, which may cause a potential selection bias and be center specific to the research results. Second, CONUT was obtained from the laboratory indicators at the time of discharge. In this study, there was no dietary information and CONUT follow-up of each patient after discharge. Due to the influence of the length of stay, CONUT may underestimate the nutritional status of patients. Third, although we demonstrated the strong prognostic power of CONUT in older adults with HFpEF, it was unclear whether CONUT can be used as a therapeutic target. To clarify the answer to this question, further longitudinal studies are needed to investigate whether nutritional intervention can improve the prognosis of heart failure.

## Conclusion

CONUT is a simple and robust prognostic marker for the prediction of readmission or all-cause mortality in older adults with HFpEF. Furthermore, compared with other objective nutritional indices, CONUT provided more powerful and practical prognostic information. Larger prospective studies are required to investigate whether nutritional interventions could contribute to favorable older adults with heart failure outcomes.

## Data Availability

All data presented in this study are reported in the article.
